# Quantitative Inspection of Remanence of Broken Wire Rope Based on Compressed Sensing

**DOI:** 10.3390/s16091366

**Published:** 2016-08-25

**Authors:** Juwei Zhang, Xiaojiang Tan

**Affiliations:** 1 College of Electrical Engineering, Henan University of Science and Technology, Luoyang 471023, China; xiaojiang_tan@163.com; 2 Power Electronics Device and System Engineering Laboratory of Henan, Henan University of Science and Technology, Luoyang 471023, China

**Keywords:** wire rope, remanence inspection, compressed sensing, wavelet filter, quantitative recognition

## Abstract

Most traditional strong magnetic inspection equipment has disadvantages such as big excitation devices, high weight, low detection precision, and inconvenient operation. This paper presents the design of a giant magneto-resistance (GMR) sensor array collection system. The remanence signal is collected to acquire two-dimensional magnetic flux leakage (MFL) data on the surface of wire ropes. Through the use of compressed sensing wavelet filtering (CSWF), the image expression of wire ropes MFL on the surface was obtained. Then this was taken as the input of the designed back propagation (BP) neural network to extract three kinds of MFL image geometry features and seven invariant moments of defect images. Good results were obtained. The experimental results show that nondestructive inspection through the use of remanence has higher accuracy and reliability compared with traditional inspection devices, along with smaller volume, lighter weight and higher precision.

## 1. Introduction

Wire ropes are widely used in industrial production, tourist cable cars, bridges, metallurgy, mining, and informal elevators. Therefore, it is important to ensure the safety of the wire ropes being used. The study of the residual strength of wire ropes is significant for developing advanced instruments that can quantitatively detect wire-rope defects [1]. Currently, the stable and safe working performance of wire ropes is of interest to more and more scholars who are interested in checking the remaining longevity of wire ropes by using online inspection devices.

Jomdecha [2] improved on equipment that was magnetized by electric current. The equipment was designed to control the strength of magnetization by adjusting the magnetized power supply or engaged loops. One special type of testing coil was designed to capture the MFL signals. An eddy current testing method [3] that used an alternating current to generate eddy current in the wire rope was proposed. A function model, which explained the relationship among defects, characteristic vectors, sensor parameters and wire ropes was established by relying on the testing data features. Raišutis [4] studied the dispersion curves of ultrasonic guided-wave spread inside wire ropes. On the basis of this research, the best and most promising receiving positions for ultrasonic guided-waves were calculated. In [5] Peng and Wang designed a visual system on the basis of gamma rays. This system focused on thick ropes used in a suspension bridge. Li et al. [6], used X-ray to detect defects in the steel core of transmission belts. They also proposed a modified threshold rules method, which captured the approximate shape of defects in the steel core.

For the detection of wire rope gaps, Wang and Tian [7] applied the analysis method of finite element to the MFL of wire ropes, and proposed an excitation method that adopted magnetic cores into a magnetic column to improve the magnetic leakage strength of gaps. A system of strong magnetic detection was designed using Hall sensors. During detection of the magnetic leakage signals of wire ropes, the air gap affected the testing accuracy, therefore, Wang et al. [8] studied the influence of different lift-off distances and different air gaps on detection accuracy and improved the structural designs of the detector and the exciter. This device inhibited the influence of lift-off variation. Li et al. [9] investigated the excitation model, established a design standard for the magnetizing structure whose theoretical size was solved through numerical solution, and used finite-element analysis to verify the theoretical size so that the final size was adjusted and determined. Some researchers adopt digital-image processing (DIP) for the MFL signals, Cao et al. [10] unrolled the MFL signals to grayscale, applied the DIP to extract the characteristics of the grayscale image, and identified the different defects. Zhang et al. [11] discussed the limits of lift-off with the digital signal processing method and designed a digital band trap to inhibit the strand waves of wire ropes. The sizes of different defects, which were processed and recognized with statistics, were described as binary images. Furthermore, Zhang et al. [12] designed a spatial filter to inhibit the strand texture of defects of grayscale image and extracted textural features of filtered defects. Finally, a BP neural network was designed and used for the quantitative identification of defects. Recently, most nondestructive testing (NDT) devices were designed using a permanent magnet as an excitation component, which excited wire rope to saturation magnetization. The MFL signals were captured by Hall sensors [10,11,12,13].

The most important aspect of a quantitative detection system is noise filtering of MFL signals. To some degree, the selected filtering algorithm would have a major effect on the quantitative inspection results. Taking into account the previously mentioned algorithm, Cao et al. [14] discussed the relationship between the temporal domain and spatial domain of electromagnetic testing signals of wire ropes, proposing a sampling theorem of the space-time signals, and the collection and processing of the space-time signals was described in detail. Tian et al. [15] combined wavelet transform (WT) and morphological transformation, and presented a morphological filtering algorithm used to inhibit the baseline drift of MFL signals. For the quantitative inspection method, Zhang and Xu [16] discussed the wavelet neural network model and weight-learning algorithm.

In this paper, GMR sensors were distributed uniformly on the circumference of the wire rope to capture the three-dimensional radial direction MFL signal of wire ropes’ residual magnetism. Compressed sensing (CS) and wavelet filtering algorithms were used to eliminate noise signals. The defect signal was translated into a two-dimensional image. For the image, the features that served as inputs for damage inspection were extracted. Experimental results show that this method can better distinguish the amount and width of broken wires and depict circumferential distribution of the defects. The device has the advantages of high detection speed, high precision, structural simplicity, as well as being lightweight, small in size, and low cost.

The paper is organized as follows: the remanence detection head device, data acquisition board and MFL imaging approach are introduced in Section 2. Section 3 focuses on noise elimination, which includes reprocessing the MFL signal and using the CSWF algorithm. The filtered MFL signal was grayed into an image that was interpolated circumferentially. Next, positioning detects and dividing negative axle waveform of defects, extracting morphological eigenvalues and invariant moments as identification vector. Section 4 presents a BP neutral network design that uses the extracted vector as inputs. Finally, the quantitative inspection of broken wires was completed. Section 5 includes comments and the discussion of this paper. Section 6 concludes the paper indicating major achievements and future scope of this work.

## 2. Acquiring MFL Signal of Remanence

**Figure 1:**
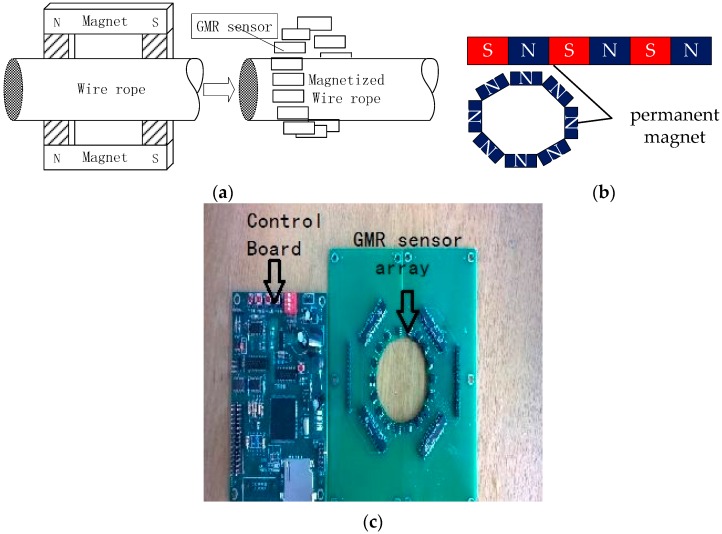
(**a**) Framework of the detection device and detection method diagram; (**b**) Excitation source; and (**c**) Signal acquisition system board.

This wire rope detection device design includes a sensor array, an excitation device and a system control board. The sensor array consisted of 18 GMR sensors, which were distributed on the rope circumferential direction to form a circle, with approximately 20° spacing between two adjacent sensors. The excitation device consists of a permanent magnet, with three small magnets placed end-to-end to form a magnetic stripe. A plurality of magnetic stripes was arranged circumferentially on the surface of the wire rope through two magnetic pole pieces in contact with the wire rope; thus, a magnetic flux loop forms between the wire rope and the excitation device (Figure 1). As shown in Figure 1a, the excitation device was used for totally magnetizing the wire rope, after which GMR sensor arrays were applied to acquire the MFL signal. The structure of the excitation device of the magnet is shown in Figure 1b. Figure 1c shows the control board and the GMR sensor array.

The capabilities of the control board include GMR array management, data acquisition, data storage, and communications. The acquisition system uses pulses, which are provided by a grating encoder, to ensure that the 18 channels acquire the MFL signal one by one. The grating encoder can send out 1024 pulses every 0.35 m, and each pulse transfers to the control board so that data acquisition for all channels would run once. Then the acquired data are stored on the SD card.

**Figure 2:**
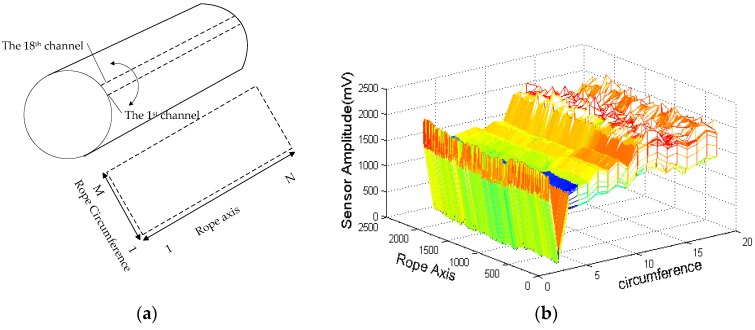
(**a**) Unrolled detection surface; (**b**) Raw data rolling image.

The raw data of 18 channels can be converted visually to form an image of the wire rope MFL. The data have equal distance in the axial direction; therefore, the rolling course only needs to unfold the data by circumferential capture order. A matrix of M × N pixels is available, where M is the number of sensors (here, the number is 18) and the N depends on the number of pulses. The unrolling processing is shown in Figure 2a, Figure 2b shows an unrolled matrix with mesh.

**Figure 3:**
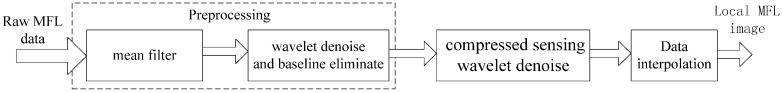
Signal preprocessing flowchart.

Serious channel imbalances and low signal-noise ratios in raw data can be seen in Figure 2b. To improve the signal-to-noise ratio and nicely capture the defect localization, the following processing steps were applied to the raw signal (Figure 3).

## 3. Data Processing

### 3.1. Signal Pre-Processing

**Figure 4:**
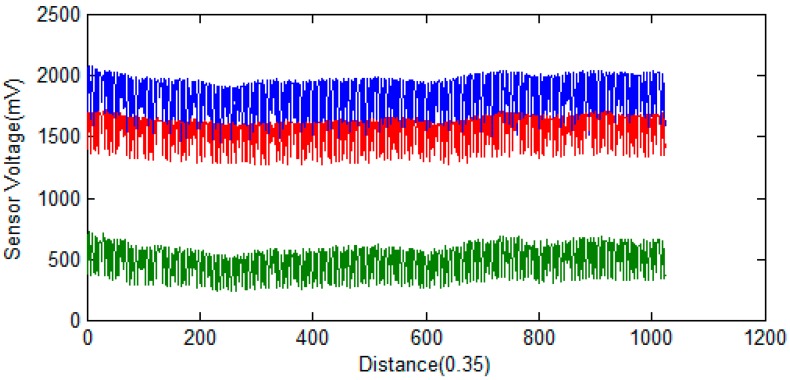
Three different sensor channels of raw MFL data.

The raw data from the GMR sensors are contaminated by various sources of noise, including high-frequency MFL noise caused by wires, lift-off variations, non-uniformity of magnetization, electronic noise, and the wire rope strands. Figure 4 shows three raw data channels. From the picture, serious channel imbalances can be clearly identified, much of which is caused by lift-off variations and non-uniform extractions, and much high-frequency noise is caused by the spiral structure of the wire ropes. To improve the signal-noise ratio and inhibit noise and channel equalization, the following pre-processing steps were applied to the raw signals.

The average filter is a simple and effective pre-processing method, implemented by meaning the neighboring MFL data [12]. This filter suppresses the influence of high frequency caused by the structure of wires and is implemented with Equation (1):

(1) $${\bar{x}}_{i,j}\;=\;\frac{1}{n}\sum_{k=m}^{m+n}x_{i,k}(i\;=\;1,2,\ldots 18,m\;=\;1,2,\ldots ,N\;-\;n)$$

where *N* is the amount of sampling, *i* is the number of channels; *j* is the current request point; *k* is the axial position of raw data; *n* is the number of points used for averaging; and *m* is the starting point of averaging.

Wavelet analysis has the characteristics of multiresolution analysis [17,18]. It can decompose a one-dimensional signal into different frequency sequences, according to the features of the signal, eliminate noisy coefficients and apply the wavelet inverse transform to get a clearer signal. By using wavelet transform on the mean signal, the baseline of the signal is a low-frequency direct current, with few defect elements in the high-frequency coefficient, so every array signal uses eight-wavelet decomposition with Equation (2):

(2) $$\left\{ \begin{array}{l} {\bar{x}}_{A_{j+1}}\;=\;{\sum h_{o}(n\;-\;2k)\bar{x}}_{A_{j}}\\ {\bar{x}}_{D_{j+1}}\;=\;{\sum h_{l}(n\;-\;2k)\bar{x}}_{A_{j}} \end{array}\right.$$

(3) $${\hat{x}}_{A_{j}}\;=\;\sum h_{o}(k\;-\;2n){\bar{x}}_{A_{j+1}}\;+\;h_{l}(k\;-\;2n){\bar{x}}_{D_{j+1}}$$

where ${\bar{x}}_{A_{j+1}}$ is the *j*-th lowest frequency coefficient; ${\bar{x}}_{D_{j+1}}$ is the *j*-th highest frequency coefficient; *h_o_*(*n*) and *h_l_*(*n*) are the decomposing filters ${\bar{h}}_{o}\left(k\right)=h_{l}\left(-k\right)$; and ${\hat{x}}_{A_{j}}$ is the approximate signal whose baseline and highest noise are eliminated.

**Figure 5:**
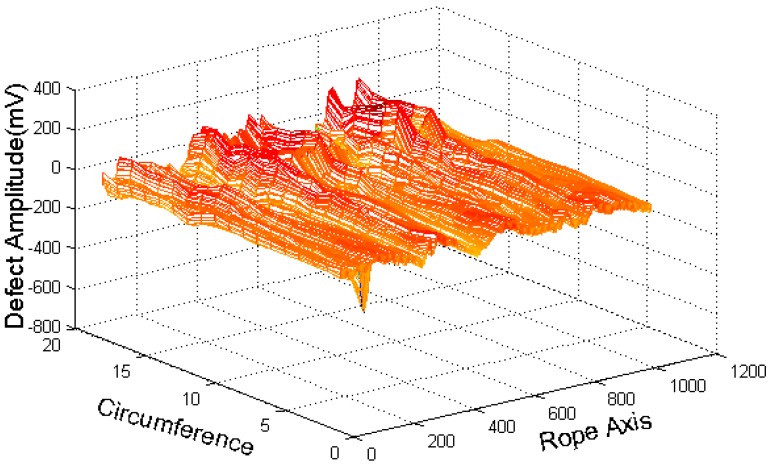
Unrolled preprocessed signals.

The available highest coefficients and lowest ones are at zero, and the two sequences of wavelet coefficients have almost no defect components. By applying Equation (3), the MFL signal without a baseline is obtained. Figure 5 shows that the MFL signal had been pre-processed. Comparison of Figure 5 and Figure 2b clearly indicates that the signal-noise ratio improves after the image pre-processing and that the high-frequency noise is inhibited lightly so that the definition of defective region is obvious.

### 3.2. Denoising Based on CSWF

The pre-processed signal ${\hat{x}}_{A_{j}}$ still contains much irregular strand waves and noise that would have a bad effect on the subsequent feature extraction and recognition processing. Because of the special structure of ropes, impurities on the rope surface, and changes to the internal structure because of twisting and uneven stress, the coercivity of the different parts of the ropes is not similar, and the strand noise does not resemble a periodic signal. Traditional digital filters cannot perform well for inhibiting these noises, but the CSWF [19,20,21] method could overcome this drawback well.

The noisy signal is not sparse in the wavelet domain. According to compressed sensing, a certain measurement matrix exists, in which the linear measurement of the wavelet coefficients is obtained [20,21]. The OMP [22,23,24] algorithm is one method for reconstructing the sparsest wavelet coefficients. The core of the OMP algorithm is that the closest matching column, which has the maximum inner product with measurement residue, is selected by greedy fashion. This column is added to the selected columns, which is then eliminated from the measurement matrix. By applying the least-squares method to all the selected columns, the approximate sparse solution was acquired, and the residue is updated. The selection process was not repeated until the iterations reached the sparsity K. Thus, the clear defect signal could be extracted with suited K.

The CSWF algorithm is as follows:

(1) For the pre-processed signal ${\hat{x}}_{A_{j}}$, the Mallat decomposition algorithm is used and the wavelet coefficients *W_j_* under each scale *j* are obtained.
(2) The appropriate random measurement matrix Φ (here is a 350 × 1024 Gaussian matrix), is selected, and the wavelet coefficients of linear measurements *y* under the measurement matrix $\Phi :y=\Phi W_{j}$ are calculated.
(3) Through the OMP algorithm, the most-sparse wavelet coefficient ${\hat{W}}_{j}$ is reconstructed; the algorithm steps are as follows:
  Step One: residue, $r_{t}{|}_{t=0}=y$, and index set, $A_{t}=\phi$ (empty set), are initialized
  For iteration, *t* is 1 to K (K is the sparse degree; here it is 8.)
  Begin
  Step Two: the inner product is calculated $\langle r_{t}●\Phi \rangle$
  Then, the column of whose inner product is the maximum in Φ is obtained: $\lambda_{t}=arg\underset{t=1\sim N}{\max}\left|\langle r_{t-1}●\Phi_{t}\rangle \right|$;
  The subscript $A_{t}=\left[A_{t-1},A_{\lambda_{t}}\right]$ is stored, and the most orthogonal column of Φ: $\Phi_{t}=\Phi_{t-1}\cup \left\{\Phi_{\lambda_{t}}\right\}$, the selected column of Φ, is set to **0**;
  Step Three: The least-squares method is used $\omega_{t}=argmin∥y-\Phi_{t}\omega_{t}{∥}_{2}={\left(\Phi_{t}^{H}\Phi_{t}\right)}^{-1}\Phi_{t}^{H}y$;
  Step Four: Approximation $y_{t}=\Phi_{t}\omega_{t}=\Phi_{t}{\left(\Phi_{t}^{H}\Phi_{t}\right)}^{-1}\Phi_{t}^{H}y$ is updated;
  The residue, $r_{t}=y-y_{t}$, is updated;
  End
(4) Using approximate wavelet coefficients ${\hat{W}}_{j}\left(A_{j}\right)=\omega_{t}$ the MFL signals are reestablished.

**Figure 6:**
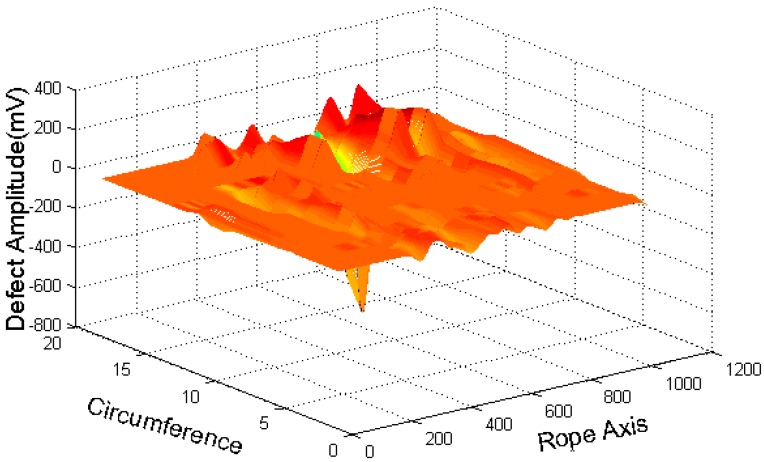
CSWF filtered three-dimensional diagram.

The CSWF algorithm eliminates the strand wave and noise, and improves the signal-noise ratio. Figure 6 shows the image processed by the CSWF algorithm.

### 3.3. MFL Image Processing

**Figure 7:**
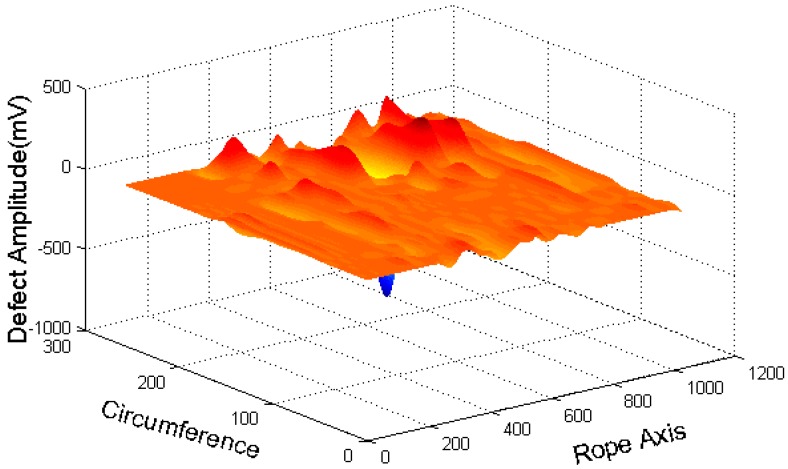
Three-dimensional diagram interpolated data.

Because of the 18 GMR sensors around the wire ropes, the captured data have low circumferential resolution, which is far below the axial resolution. Then, the clear signals are interpolated to improve the circumferential resolution. Figure 7 shows the image with the interpolated data.

#### 3.3.1. Defect Image Extraction

**Figure 8:**
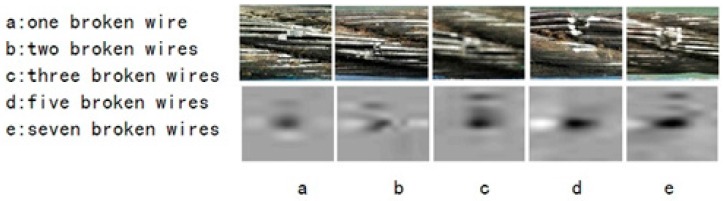
Photos (**above**) and local MFL image (**below**).

Before defect characteristic extraction, the MFL defect photos need to be positioned and segmented. The local modulus maxima algorithm is adopted to locate the defects. From experience, the produced minimum artificial defect is more than 100 mV, so in this paper, 100 mV was set to be a threshold, used for judging whether the part is defective. As shown in Figure 8, various defects can be detected. In the captured image, different broken wires form different defect photos, which show the main power concentrated on broken wires, centered in a cone-like recess, which is also shown in Figure 6. Five different broken wires photos and local MFL images were stretched between 0**–**255 and, the size of local MFL is 200 × 200 pixels.

#### 3.3.2. Defect Characteristic Exactions

By the procedure given, a local MFL image of the localized defects is presented, and the geometric features and moment invariants of the MFL image can be used to identify defects. The geometric features describe the basic shape of object, and the moment invariant is the average description of area gray distribution, which is calculated by all points in the area and is less susceptible to noise. In total, ten characteristics of the MFL image were selected, including the equivalent area, the slenderness ratio, and the circularity and first- to seven-order moment invariants (Φ_1_–Φ_7_).

1. Basic Description of Image Shape

Geometric features were calculated by brief description of features, such as: area (*S*), perimeter (*L*), major axis (*L*_1_), and minor axis (*L*_2_). All these descriptions are as follows.

The area of defect image is:

(4) $$S\;=\;\sum_{(x,y)\in R}I$$

where *R* is the set of points in the defect region; *S* is the amount of high-value in binary image; and *I* is the binary image.

**Figure 9:**
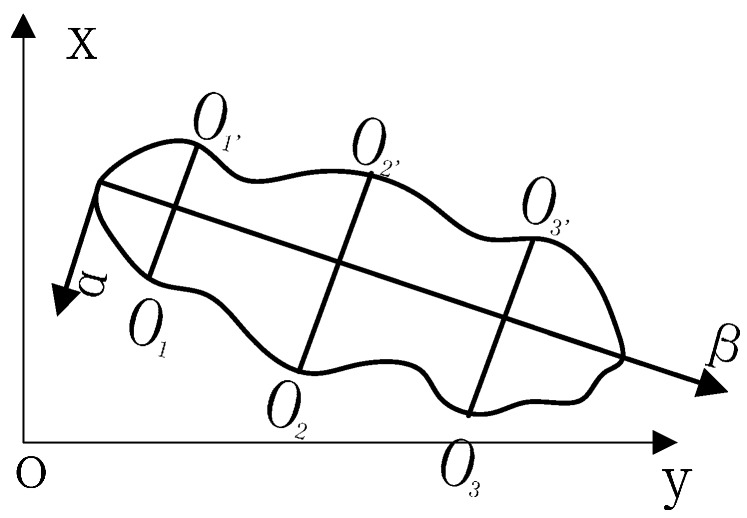
*L1* and *L2* sketch map.

The defect perimeter is the total length of the outer boundary, which can be expressed by the sum of the distance between adjacent pixels. If the number of pixels of the outer boundary is *n*, its chain code, *c_i_* is followed by *c*_1_, *c*_2_, *c*_3_, …, *c_n_* and the perimeter can be presented as follows:

(5) $$L\;=\;\frac{\sqrt{2}\;+\;1}{2}n\;-\;\frac{\sqrt{2}\;+\;1}{2}\sum_{i=1}^{n}{\left(-1\right)}^{c_{i}}$$

where *L*_1_ is defined as the maximum distance between any two points in the outer boundary, and *L*_2_ is defined as the longest straight line which is vertical to *L*_1_, as shown in Figure 9.

Assuming two random points are present in the outer boundary, *α*_1_(*x*_1_,*y*_1_) and *α*_2_(*x*_2_,*y*_2_). The endpoints of the vertical line are *v*_1_(*m*_1_,*n*_1_) and *v*_2_(*m*_2_,*n*_2_) to *L1*. The *L*_1_ and *L*_2_ are calculated as follows:

(6) $$\left\{ \begin{array}{l} L_{1}\;=\;\max(\sqrt{{\left(x_{1}\;-\;x_{2}\right)}^{2}\;+\;{\left(y_{1}\;-\;y_{2}\right)}^{2}})\\ L_{2}\;=\;\max(\sqrt{{\left(m_{1}\;-\;m_{2}\right)}^{2}\;+\;{\left(n_{1}\;-\;n_{2}\right)}^{2}}) \end{array}\right.\;s.t.\;(x_{1}\;-\;x_{2})(m_{1}\;-\;m_{2})\;+\;(y_{1}\;-\;y_{2})(n_{1}\;-\;n_{2})\;=\;0$$

2. Characteristic Descriptions of Shape

Because of the different sizes of wire rope and lift-off variations, the detected defect area, perimeter, and length-width are not similar in the same case. Therefore the basic description is not taken as the recognition features. Nevertheless the equivalent area, which is the ratio of area and perimeter, represented by *G*, is taken as the recognition feature:

(7) G = S/L

where the equivalent area *G* reflects the surrounded region of defects by unit perimeter. If the shape of the defect is circular, the ratio of area and perimeter is the minimum.

Slenderness ratio *F* is defined as the ratio of the major axis *L*_1_ and the minor axis *L*_2_:

(8) $$F\;=\;L_{1}/L_{2}$$

The slenderness ratio reflects the shape of the defects. It is a sensitive parameter of the circular boundary. When the defect shape forms a circle, the long and short diameters are relatively close, and the *F* value is close to 1. The greater the ratio of the long axis to the short axis, the slimmer the shape of the defect.

The circular degree of the image is the complexity degree of the area shape measured on the basic of the area and perimeter. Its mathematical expression is as follows:

(9) $$e\;=\;4\pi S/C^{2}$$

where *e* is the circularity of the defect; *S* is the area; and *C* is the perimeter.

When the object region is circular with the radius, *r*, its area $S=\pi r^{2}$ and its perimeter is C= 2πr. That is, its circularity e = 1. This characteristic reflects the complexity of the shape in the area. If the shape is closed to a circle, *e* is bigger, and the maximum is value 1. If the shape is more complex, *e* is closer to 0.

3. Characteristics of Invariant Moment

Invariant moments are established on statistical analyses of the gray distribution of the target area, a sort of statistical description on average. It describes the overall characteristics of an object from a global view, thus, it is less susceptible to noise and would not change with the translation, rotation and scale of the image [25]. For this paper, we chose to describe the shape characteristics of the defect image.

Given an image *f*(*x,y*), if it is piecewise continuous, with a limited non-zero number available on the plane, its varied order exists. The two-dimensional (*p + q*) order moment of *f*(*x,y*) is defined as [25]:

(10) $$m_{pq}\;=\;\sum_{x}\sum_{y}x^{p}x^{q}f(x,y)$$

The value of moments, *m_pq_*, will change when (*x,y*) is translated. To reduce and eliminate unfavorable effects, the central moments are defined as:

(11) $$u_{pq}\;=\;\sum_{x}\sum_{y}{\left(x\;-\;\bar{x}\right)}^{p}{\left(y\;-\;\bar{y}\right)}^{q}f(x,y)$$

where $\bar{x}$ and $\bar{y}$ are the center of gravity, defined as:

(12) $$\bar{x}\;=\;m_{10}/m_{00},\;\bar{y}\;=\;m_{01}/m_{00}$$

On the basis of the defined central moments, the seven invariant moments are defined as follows [25]:

(13) $$M_{1}\;=\;u_{20}\;+\;u_{02}$$

(14) $$M_{2}\;=\;{\left(u_{20}\;-\;u_{02}\right)}^{2}\;+\;4u_{11}^{2}$$

(15) $$M_{3}\;=\;{\left(u_{30}\;-\;3u_{12}\right)}^{2}\;+\;{\left(3u_{21}\;+\;u_{03}\right)}^{2}$$

(16) $$M_{4}\;=\;{\left(u_{30}\;+\;u_{12}\right)}^{2}\;+\;{\left(u_{21}\;+\;u_{03}\right)}^{2}$$

(17) $$\begin{array}{l} M_{5}\;=\;{\left(u_{30}\;-\;3u_{12}\right)}^{2}\;+\;(u_{12}\;+\;u_{30})[{\left(u_{30}\;+\;u_{12}\right)}^{2}\;-\;3{\left(u_{21}\;+\;u_{03}\right)}^{2}]\;+\;\\ \;(3u_{21}\;-\;u_{03})(u_{21}\;+\;u_{03})\left[3{\left(u_{30}\;+\;u_{12}\right)}^{2}\;-\;{\left(u_{21}\;+\;u_{03}\right)}^{2}\right] \end{array}$$

(18) $$M_{6}\;=\;(u_{20}\;+\;u_{02})[{\left(u_{30}\;+\;u_{12}\right)}^{2}\;-\;{\left(u_{21}\;+\;u_{03}\right)}^{2}]\;+\;4u_{11}(u_{30}\;+\;u_{12})(u_{21}\;+\;u_{03})$$

(19) $$\begin{array}{l} M_{7}\;=\;\left(3u_{21}\;-\;u_{03}\right)\left(u_{30}\;+\;u_{12}\right){\left(u_{30}\;+\;u_{12}\right)}^{2}\;-\;3{\left(u_{21}\;-\;u_{03}\right)}^{2}\;+\;\\ \;(3u_{12}\;-\;u_{30})\left(u_{21}\;+\;u_{03}\right)[3{\left(u_{30}\;+\;u_{12}\right)}^{2}\;-\;{\left(u_{21}\;+\;u_{03}\right)}^{2}] \end{array}$$

Equations (10) to (19) are implemented in the defects image. Seven invariant moments are calculated as the characteristic vectors of the image. In this paper, we selected four different wire rope structures as detection examples. They were 6 × 19, 6 × 36, 6 × 37, and 7 × 27. According to the characteristic extraction above, parts of vectors of one wire rope are listed in Table 1.

## 4. Quantitative Recognition

The BP neural network [16,26,27] is the most studied and widely used method in target recognition. A three-layer neural network can approach any nonlinear function. For this paper, the BP neural network was selected for defect recognition. A BP neural network model was built, including an input layer, a hidden layer and an output layer. The ten extracting characteristic vectors from the former processing are taken as the input of the neural network. The designed BP has a single output, so the structure of the designed BP neural network is 10 × *N* × 1 (*N* represents the number of hidden nodes). The function “tansig” was selected as the transfer function of the hidden layer and “logsig” one was the transfer function of the output layer. In this paper, the output of the neural network is the percentage of broken wire. For example, a wire rope of 9 × 19 structure with a defect of concentrated broken wires with the amount of three, because of the output range, the characteristic vectors of defect need to be normalized to [0, 1], so the output would be 0.175. In the experiment, four kinds of wires were chosen including 9 × 19, 6 × 36, 6 × 37 and 7 × 27, and their diameters were 22, 25, 28, 30, 31, 32, and 34 mm, separately.

**Figure 10:**
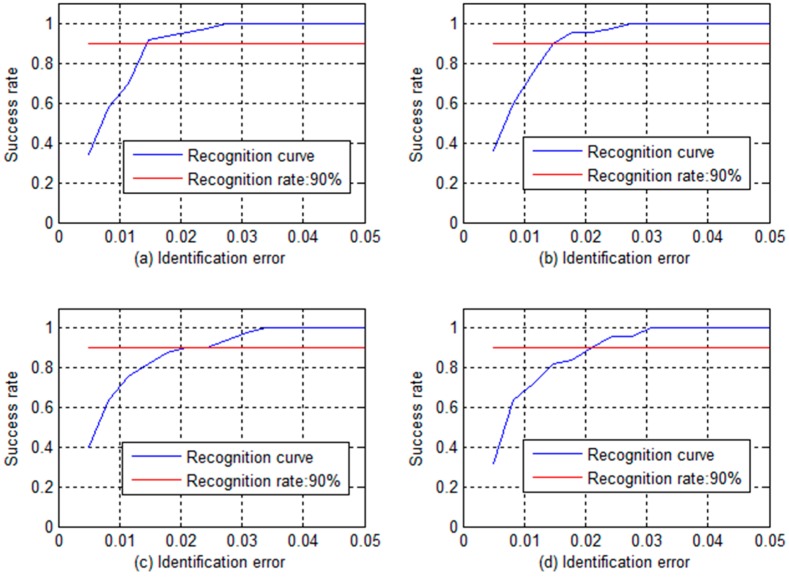
Different hidden nodes recognition graphs: (**a**) Identification ratio graph of 21 hidden layer nodes; (**b**) Identification ratio graph of 24 hidden layer nodes; (**c**) Identification ratio graph of 27 hidden layer nodes; and (**d**) Identification ratio graph of 30 hidden layer nodes.

**Figure 11:**
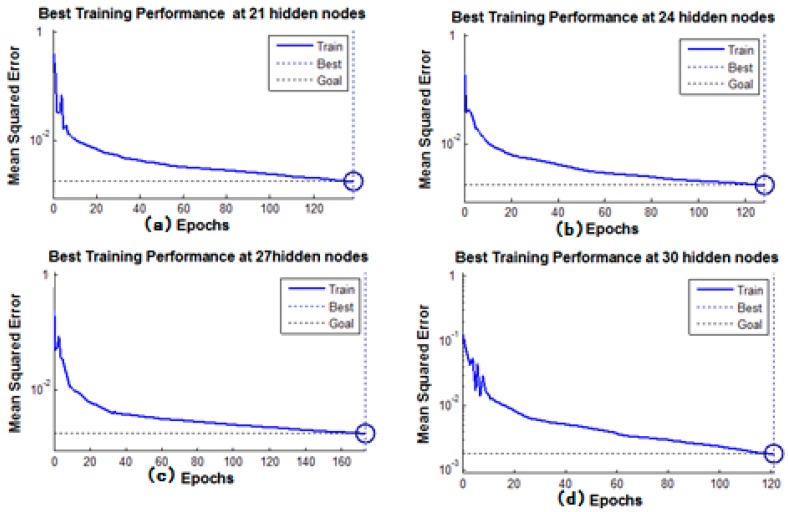
Training performance graphs for different hidden layer numbers: (**a**) 21 hidden nodes; (**b**) 24 hidden nodes; (**c**) 27 hidden nodes; and (**d**) 30 hidden nodes.

There were 105 samples of various defects in the experiment. Of the samples, 55 were randomly selected as training samples, and the others were selected for testing. The BP neural network was built by using *MATLAB*. The training error of BP was set so low that the training network had great convergence. Various hidden layer nodes of the network had different ratio of inspection, part of them is shown as Figure 10, whereas the network training performance graph of the different hidden nodes is shown in Figure 11. Table 2 presents the training results and test samples in quantitative inspection of the percentage of broken wires. In different hidden layers, the iteration times and training time vary significantly. The best performance of the BP network was seen when the number of hidden layers was 21 and the recognition error of training samples was less than 1.075%. For test samples, when the allowable identification error is 1.5%, the success rate is as high as 94%, but the recognition error is less than 2.571%.

## 5. Comment and Discussion

With GMR sensors areuniformly distributed around the wire rope, the MFL of remanence in the vicinity of the wire rope is captured, and the raw data are unrolled to form the MFL image, as shown in Figure 2. The channel imbalance and high-frequency noise are serious in the raw signals. The pre-processing based on wavelet transfer is used for suppressing the influence which is caused by lift-off variation, non-uniformity of magnetization and the wires. Comparison of Figure 5 with Figure 2b indicates that the channel consistency is greatly improved and that high-frequency is inhibited. Then the CSWF method is presented in this paper to inhibit the noise further. Figure 6 shows that the noise is almost rejected. Furthermore, the data interpolation is introduced to improve the circumferential resolution of the MFL image, and the local MFL image is scale normalized to the size of 200 × 200 pixels. Three characteristics of shape and the seven-order invariant moment of the MFL image are used as input for BP networks for the quantitative inspection of wire rope defects. Network performances vary greatly in different number of hidden layers. The best performance is met when the number of hidden layer nodes is 21. Its recognition is as high as 94%, and recognition error is 1.5%.

## 6. Conclusions

The MFL of remanence inspection equipment with GMR sensor arrays has been designed to overcome the limitations of traditional equipment. Both the circumferential and the axial distributions of defects are obtained, which improves the defect-detection resolution. The MFL image consists of 18-channel data from the GMR sensor array. Signal-to-noise ratio is further improved by CSWF. Circumferential resolution is improved by interpolation. Three description characteristics of shape and seven-order invariant moments are extracted as features of the MFL image. They are utilized as the inputs of the BP networks to classify different defects. The results show that it is possible to implement the quantitative inspection of broken wires by utilizing the remanence of wire rope, and the CSWF method can inhibit noise sufficiently to receive clear MFL signals. For future work, more characteristics should be extracted as BP network input, which would change the recognition style and correct a greater range of defects to improve the generalization ability of network and the performance of detection systems.

## Figures and Tables

**Table 1:** Parts of the characteristic vectors of the defects.

Broken Wires	*G*	*F*	*e*	Φ_1_	Φ_2_	Φ_3_	Φ_4_	Φ_5_	Φ_6_	Φ_7_
1	4.22	0.864	0.647	6.69 × 10^10^	4.48 × 10^21^	2.88 × 10^20^	2.35 × 10^20^	4.02 × 10^37^	−2.26 × 10^28^	6.27 × 10^39^
2	6.06	0.392	0.537	6.71 × 10^10^	4.51 × 10^21^	2.66 × 10^20^	2.36 × 10^20^	−2.76 × 10^42^	−4.03 × 10^29^	−1.24 × 10^42^
3	11.9	0.935	0.623	6.70 × 10^10^	4.49 × 10^21^	7.26 × 10^21^	2.57 × 10^21^	3.83 × 10^42^	2.51 × 10^28^	−4.9 × 10^42^
4	19.6	1.150	0.642	6.58 × 10^10^	4.32 × 10^21^	2.11 × 10^21^	1.51 × 10^21^	−2.01 × 10^43^	−6.24 × 10^30^	3.15 × 10^43^
5	7.15	0.364	0.499	6.58 × 10^10^	4.33 × 10^21^	5.52 × 10^21^	5.62 × 10^21^	−1.19 × 10^44^	−7.64 × 10^30^	−7.68 × 10^42^
7	1.64	0.811	0.592	6.65 × 10^10^	4.42 × 10^21^	6.67 × 10^21^	4.95 × 10^21^	−3.16 × 10^43^	7.42 × 10^29^	−9.84 × 10^43^

**Table 2:** Performance of the BP network in different hidden layers.

Hidden Layer Number	Iteration Time (s)	Maximum Error for Training Set (%)	Maximum Error for Test Set (%)	Train Sample Number
21	138	1.353	2.521	55
24	128	1.606	2.734	55
27	173	1.479	4.211	55
30	121	1.075	2.732	55
